# SSWAP: A Simple Semantic Web Architecture and Protocol for semantic web services

**DOI:** 10.1186/1471-2105-10-309

**Published:** 2009-09-23

**Authors:** Damian DG Gessler, Gary S Schiltz, Greg D May, Shulamit Avraham, Christopher D Town, David Grant, Rex T Nelson

**Affiliations:** 1 University of Arizona, 1657 E. Helen St., Tucson, AZ 85721, USA; 2 National Center for Genome Resources, 2935 Rodeo Park Drive East, Santa Fe, NM 87505, USA; 3 Cold Spring Harbor Laboratory, One Bungtown Road, Cold Spring Harbor, NY 11724, USA; 4 The J. Craig Venter Institute, 9704 Medical Center Drive, Rockville, MD 20850, USA; 5 USDA-ARS-CICGR and Department of Agronomy, Iowa State University, Ames IA, 50011, USA

## Abstract

**Background:**

SSWAP (**S**imple **S**emantic **W**eb **A**rchitecture and **P**rotocol; pronounced "swap") is an architecture, protocol, and platform for using reasoning to semantically integrate heterogeneous disparate data and services on the web. SSWAP was developed as a hybrid semantic web services technology to overcome limitations found in both pure web service technologies and pure semantic web technologies.

**Results:**

There are currently over 2400 resources published in SSWAP. Approximately two dozen are custom-written services for QTL (Quantitative Trait Loci) and mapping data for legumes and grasses (grains). The remaining are wrappers to Nucleic Acids Research Database and Web Server entries. As an architecture, SSWAP establishes how clients (users of data, services, and ontologies), providers (suppliers of data, services, and ontologies), and discovery servers (semantic search engines) interact to allow for the description, querying, discovery, invocation, and response of semantic web services. As a protocol, SSWAP provides the vocabulary and semantics to allow clients, providers, and discovery servers to engage in semantic web services. The protocol is based on the W3C-sanctioned first-order description logic language OWL DL. As an open source platform, a discovery server running at  (as in to "swap info") uses the description logic reasoner Pellet to integrate semantic resources. The platform hosts an interactive guide to the protocol at , developer tools at , and a portal to third-party ontologies at  (a "swap meet").

**Conclusion:**

SSWAP addresses the three basic requirements of a semantic web services architecture (*i.e*., a common syntax, shared semantic, and semantic discovery) while addressing three technology limitations common in distributed service systems: *i.e*., *i*) the fatal mutability of traditional interfaces, *ii*) the rigidity and fragility of static subsumption hierarchies, and *iii*) the confounding of content, structure, and presentation. SSWAP is novel by establishing the concept of a canonical yet mutable OWL DL graph that allows data and service providers to describe their resources, to allow discovery servers to offer semantically rich search engines, to allow clients to discover and invoke those resources, and to allow providers to respond with semantically tagged data. SSWAP allows for a mix-and-match of terms from both new and legacy third-party ontologies in these graphs.

## Background

Biology is rapidly becoming an information science. This is driving the need for machines to become increasingly sophisticated in finding, assimilating, and integrating information. Sharing information is at the core of informatics. For bioinformatics, this has been recognized in its various forms since its inception [1-5]. But what was initially seen as an issue of the setting of standards for sharing data, has grown into a mature assessment that distributed, decentralized, possibly ephemeral, resources are now the fabric of the informatic landscape. Navigating this landscape requires technologies that far exceed simply establishing connectivity and sharing data via a common format. The fact that interconnectivity and interoperability protocols and middleware such as FTP (1971), Telnet (1973), Ethernet (1973), TCP/IP (1974-83), SMTP (1982), Gopher (1990), HTTP (1990), and CORBA (1991), have been with us for nearly 40 years, yet the level of integration we want in informatics remains elusive, shows that broad, interoperability standards *per se *may be necessary but are not sufficient for integration.

The requirements for (inter)connectivity, interoperability, and integration, form a dependency stack, whereby transitively the former satisfy necessary but not sufficient conditions for the latter. Interconnectivity addresses those technologies necessary for two or more computers to create a network, such that they can be said to be "connected" and able to send and receive bits without loss of information. Interoperability implies a two-way exchange based on common protocols, and uses interconnectivity in an application-specific manner to build productivity. Integration builds upon this with a synthetic attribute, whereby distributed information is aggregated and assimilated either physically or virtually so as to create a whole greater than the sum of the parts.

As we move from interconnectivity to integration, the informatic problem matures from one of predominately specifying a suitable protocol and *syntax *to one of developing and deploying an expressive *semantic *(from the Greek *semaino *"to mean"; *semantikos *"significant"). Alternatively, we can see this as moving from a framework with an explicit syntax and implicit semantics, to one where both the syntax and semantics are now explicit. The requirement for an explicit semantics is driven as much by the increasing sophistication of science as by the need to address current technology limitations. For example, in the area of bioinformatics, scientists *want *the synthetic whole generated from an integration of genomic and functional data, but they *will *generate the underlying data only from within the fractured sociological infrastructure of separate disciplines of scientific thought, research establishments, and funding programs. Because data is generated in different semantic spaces, integrating it requires knowledge of the data's context and suitability-for-purpose. In a low-throughput environment, where we connect resources and integrate data on a case-by-case basis with human intervention, the semantics may be implicit; *i.e*., evident to humans by reading documentation and applying appropriate judgments on use. But in a high-throughput environment, where we want machines to find distributed information, to assimilate, and integrate it automatically, we are forced to raise semantics to the level of explicit statements available for computation.

There are notable efforts to address these and related issues. EMBL-EBI provides web services for over two dozen operations including sequence similarity, multiple sequence alignment, data retrieval, and others [6-9]. Many EMBL-EBI web services use the industry-standard technologies of WSDL (Web Service Definition Language) documents to describe services and SOAP (Simple Object Access Protocol) as the protocol for interoperability. EMBL-EBI also support REST (REpresentational State Transfer) interfaces for some services. In a similar manner, NCBI offers web services using standard WSDL and SOAP descriptions and protocols. These web services can be engaged via the REST-like, URL-based eUtils services that are essentially HTTP wrappers to the Entrez query system [10]. Indeed, EMBL EBI and NCBI are just two of many bioinformatic web services available (*e.g*., [11]). To discover and engage such services, one needs to either peruse the unstructured information on web pages and within the published literature, or use a discovery service specific for web services. For example, BioCatalogue [12] provides a Google-like search service for web services, while BioMoby [13] provides an XML-based namespace and object ontology that both enables discovery and invocation for BioMoby-compliant services. A limitation of WSDL and SOAP is that their syntactical rules of engagement do not allow for machines to readily determine the semantics of the underlying data and services. Efforts have been made to address this. One approach, as taken by the myExperiment project is to encapsulate the underlying service details in a higher-order integrative layer. myExperiment combines social networking with scientific workflows [14], thereby emphasizing not the web services per se, but the end product of their thoughtful integration. Moving to a state where machines can better contribute to higher level integration has been slow coming, in part because steady progress in implementing an infrastructure that allows machines to discover services and assess their suitability-for-purpose in a fully automated manner presupposes a formal logic over web resources (*e.g*., [15]). It has only been relatively recently that the tripartite of syntax, semantics, and logic is available in a web standard, such as the W3C standard of OWL (Web Ontology Language [16-19]).

In this paper, we examine high-throughput integration (the "integration equation") by first highlighting three technology limitations that are hindering current solutions; we then describe the SSWAP architecture as an approach to addressing these limitations by delivering on the syntax/semantics/logic stack; lastly we point to its deployment as a platform in the Virtual Plant Information Network (VPIN).

### Three current technology constraints limiting integration over the web

We identify three current technology limitations that are hindering solutions to the "integration equation." They are the:

1. *fatal mutability of traditional interfaces *- the problem where if data and service providers change their interface signatures, client code depending on that signature fails *en masse*. This has the undesirable property that the more clients engage an interface (*i.e*., the more widely it is adopted), the less flexibility providers have in evolving it. For example, consider a provider's interface that is simply a URI (Uniform Resource Identifier) expected to be dereferenced by a HTTP GET: http://www.myProvider.org/myResource?geneName="CDC40". If the provider now changes the query substring to http://www.myProvider.org/myResource?locusName="CDC40", then scripts and programs expecting the former syntax fail. HTTP as a protocol *per se *and REST as an architectural style offer no protection from this *en masse *failure; it would be up to the provider to address backwards compatibility by, for example, supporting both URIs concurrently. This fuels rigidity (the inability of software to change to meet new demands) and fragility (the propensity of software to fail, often in multiple and seemingly unrelated places, under changes). The problem occurs because the binding, *i.e*., the synchronization of client code to the host's signature, is traditionally established both syntactically and semantically when the client code is written, rather than when it is invoked. What we would like is an architecture that supports late binding, whereby signature validation is delayed until transaction time, preferably within a logical framework whereby clients and hosts could negotiate and assess suitability-for-purpose.

2. *rigidity and fragility of static subsumption hierarchies *- the problem where changing the properties of a class near the root of an inheritance hierarchy (an ontology) redefines subsumption criteria for the entire sub-tree. This has the undesirable property that nodes near the root, which were built when the system was least evolved, are the least able to change without generating cascading repercussions. The core issue for semantic web services is that static asserted subsumption hierarchies--in contrast to dynamic subsumption hierarchies calculated closer to transaction time--do not lend themselves well to changes in application, while often leading to a confounding of concepts with their data model: the extreme (yet common) case being when concepts have no explicit properties, meaning that attributes are implied solely from the class' position in the subsumption hierarchy. For example, the statements '*M subClassOf V*; *V subClassOf E*' implies that any individual of type *M *(*e.g*., *Magnoliophyta *[flowering plants]) has all the necessary and sufficient properties of type *V *(*e.g*., *Viridiplantae *[green plants]) and may be more; and similarly *V *to *E *(*e.g*., *Eukaryota *[eukaryotes]). Yet without explicitly delineating those properties (*i.e*., codifying them to make them available to reasoners), machine reasoners are limited in what they can infer from the ontology. This is immediately apparent in many of the Open Biomedical Ontologies, for example, in the 20,000+ node Gene Ontology [20]. The Gene Ontology has separate ontologies for Biological Process, Molecular Function, and Cellular Component exactly because subsumption claims on one topic--*e.g*., Biological Process--are of little value when organizing knowledge on another topic, *e.g*., Molecular Function. The Gene Ontology's well-deserved success rests largely on its authoritative subsumption hierarchy and community acceptance of the implied underlying logic [21]. For high-throughput integration--particularly cross-ontology integration--the problem of asserted *vs*. derived subsumption becomes acute when the properties underlying the justification of the subsumption statements are not themselves defined within the ontology (as is often the case). In that situation, the subsumption hierarchy stands as essentially a static statement of axiomatic relationships. One possible solution to this problem is to deploy ontologies with a greater emphasis on properties [22-24] and push subsumption determination closer to transaction time.

3. *confounding content, structure, and presentation *- the problem where the information payload--the data itself--is entangled with its data structure and/or the presentation layer and implicit behaviors of the presentation software. HTML and many applications exploiting the hierarchical nature of XML suffer from this type of entanglement. This has the undesirable property that the data of value to the client may be difficult or essentially impossible to generically parse from the data delivered by an arbitrary provider, thereby crippling machine-automated disentanglement. When data is buried deep in XML hierarchical structures, or available only as "hidden" side-effects of semantically opaque SOAP signatures, or encoded as a combination of images and tables in HTML, the client is restricted in the utility it can extract from the data, even if such restrictions are not the intent of the provider. What we would like is an architecture that supports a clean separation of content, structure, and presentation, whereby a provider's dissemination of the data imposes minimal side-effects on the client's suitability-for-purpose.

No protocol completely address all these limitations in all scenarios. But in the protocol we describe here we make progress on them specifically by grounding a new protocol in a formal logic. Thus the fatal mutability of interfaces is addressed by designing a protocol that allows for cached or transaction time reasoning for assessment of what the service is offering and what it returns. Similarly, by allowing services to describe their offerings using classes and properties, it is backwards-compatible with deep subsumption hierarchies while also enabling just-in-time subsumption determination based on common properties instead of explicit subclass assertions. Lastly, the protocol encapsulates the semantic description of services and data from the presentation layer using non-hierarchical RDF (Resource Description Framework [25,26]), thereby helping to separate content, structure, and presentation.

### Syntactical, Semantic, and Discovery Requirements

An architecture that addresses the above challenges would go a long way to achieving the robustness, evolvability, and scalability that are required for high-throughput integration [13-15]. In light of these challenges, we were impressed with the overwhelming success of the web; its stateless, method-sparse, document-based architecture demonstrating many desirable properties of robustness, evolvability, and scalability. Our solution was to design an architecture that mimicked much of what worked for the web; in particular, a document-based architecture with explicit delineations of data and its contextual relationships. This leaves the manipulation of the data's "raw value" to any particular technology of the day, allowing the system to evolve as new technologies are developed. A technology assessment phase in an earlier related project called Semantic MOBY (an independent arm of BioMOBY) led to the choice of RDF and OWL DL as the underlying enabling technologies.

These considerations essentially recast any solution to the "integration equation" from being one of specifying a syntax and messaging layer used to connect clients and providers, to being one of providing clients and providers a way to *describe *their queries and data, find each other on the web, and engage semantic negotiation to determine suitability-for-purpose at transaction time. This requires a solution to satisfy a set of three basal requirements; *viz*., to:

• *deploy a common syntax *- that is, allow clients and providers to engage each other under shared syntactical rules. Currently, the GET query strings to major web-based biological information resources such as Entrez , Gramene , and LIS  all have differing and idiosyncratic syntaxes, thereby making interoperability consist of one-off scripts that are inherently non-scalable;

• *develop a shared semantic *- that is, allow machine-discernable meaning so clients can request the same conceptual object or service from different providers. For example, many providers offer equivalent DNA sequences or sequence comparison algorithms, yet scripts cannot compare and contrast the offerings without low-throughput, case-by-case customization. An infrastructure for semantic negotiation is needed; especially one that is cognizant of the sociological influences of achieving a shared semantic;

• *implement a discovery server *- that is, allow clients to find providers based on the semantics of their data and services. Specifically we introduce the capability of semantic searching as defined below. As built upon a common syntax and semantic amenable to a formal logic, this is the necessary condition for scalable integration.

Our design goals are to address the three technology limitations listed earlier while satisfying the three requirements listed above. We found both current-day pure-play semantic web and pure-play web service solutions lacking in this regard--the former because of its lack of web service protocols and the latter because of its lack of a formal semantics amenable to reasoning. Cognizant of this, our design approach is to deeply embed the semantic web "philosophy" to the application of web services. We describe here a new protocol to do this. What follows is a document-based design that uses a W3C-compliant middle-layer for a semantically rich encoding of data and service descriptions.

## Implementation

### SSWAP: A Simple Semantic Web Architecture and Protocol

Architecturally, SSWAP posits the existence of four actors on the web: *i*) providers: web sites offering resources--essentially web services for data retrieval or remote algorithm execution; *ii*) clients: users of web resources. Providers may also be clients, just as traditional web servers may both request and supply web pages; *iii*) a discovery server: a non-exclusive, web broker that helps clients find providers based on the semantic qualifications of desired data or services (collectively referred to as resources). The architecture does not require that there be any exclusive or specially authorized discovery server; *iv*) ontologies: terms used by all actors to communicate in a semantically consistent manner. As will become evident, ontological definitions may be hosted term-by-term by anyone on the web, so any web server may act as an ontology server. In many cases providers may act as ontology servers for concepts that are specific to their offerings. This basic model of providers, clients, and discovery servers (search engines) is purposely analogous to our common understanding of the World Wide Web as it operates today.

SSWAP is deeply based on RDF (Resource Description Framework [25,26]), both in its use of RDF as a technology and also in RDF's conceptual model of presenting information in the form of entities and relationships. RDF allows semantics to be expressed via a series of subject-predicate-object assertions (an 'RDF-triple'). Assertions simply state that some "thing" (the subject) has some relationship (the predicate, also called a 'property') to something else (the object). A series of subject-predicate-object triples creates a network of assertions that can be represented as a graph. RDF may be serialized in a number of different formats; the W3C specifies RDF/XML as the recommended messaging layer. In this manner RDF and RDF/XML set the basic underlying syntax for anyone to say anything about anything [27]. OWL (Web Ontology Language [16-19]), and specifically OWL DL and the newer OWL 2.0 variants, is the W3C recommendation of how to express a first-order description logic for the web. OWL is based on RDF. OWL introduces reserved classes and predicates with a formal semantic so that machines can reason over statements and come to conclusions. OWL DL is mappable to an underlying first-order Description Logic (DL) with guarantees of completeness and decidability. This provides a precise and explicit semantics which enables DL reasoning. Theoretically, the ability to reason should alleviate the need for static "is a" relationships, because subsumption (*i.e*., subclass) relationships can be computed dynamically from examining an aggregation of properties. In practice, just-in-time or even offline reasoning presents computational challenges even for some non-worst case scenarios, though experience shows these challenges are manageable in a wide variety of cases. Indeed, simple subsumption can be determined without DL reasoning, but adding DL support allows restrictions on cardinality and complex classes built upon union and intersection operations that are useful in real-world applications. An important advantage to this approach is that instead of needing to declare all "is a" relationships directly at design time one can state properties about subjects (*i.e*., subject-predicate-object assertions) as the evidence so supports, and thereby allow others to use property aggregation dynamically at transaction time. This has a natural extension to ontology alignment.

**Figure 1:**
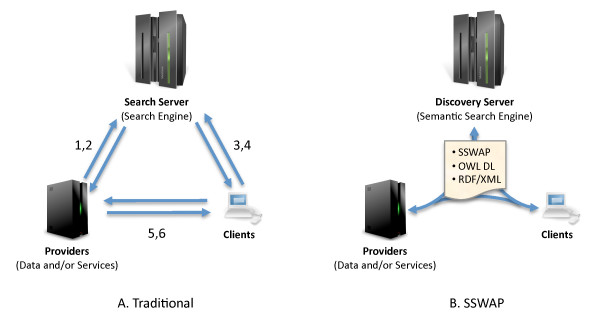
**Traditional *vs*. SSWAP Information Flow**. A) In a traditional model, different formats and technologies are used for registering/publishing (1,2), discovering (3), discriminating (4), invoking (5), and returning results from (6) services. B) In SSWAP, all actors operate on mutable, SSWAP-compliant, OWL DL graphs serialized in RDF/XML. Actors exchange graphs for description, discovery, querying, invocation, and response. All graphs are variations on the structured canonical protocol. An HTTP GET on a SSWAP semantic web service returns the SSWAP-compliant, OWL DL description of the service; an HTTP POST to the same URI with the query data in the body of the message (or HTTP GET with the graph serialized in the URI query string) invokes execution; the response is a similar OWL DL graph, but with explicit mappings of the query data to the return data. In all cases URIs to the data can be used instead of embedding the data itself.

SSWAP establishes a small set of reserved classes and predicates to allow OWL to be used for semantic web services. This is the protocol itself; essentially a light-weight SSWAP ontology designated in this paper with the prefix '*sswap:*' and available at . In SSWAP, all actors operate on a SSWAP-compliant, OWL DL graph. The graph uses the SSWAP protocol  to express the relationship between a semantic web service and its data. This architecture (Figure 1) is different from traditional provider/client/search-engine models. In traditional systems, different technologies are used to describe services, to discover services, to engage them, and to handle the result. In SSWAP, all actors work from the same, mutable graph; a graph that always adheres to the same canonical structure. This establishes both a common syntax and skeleton structure across all actors and activities and yields benefits in terms of shared parsing constructs and re-usable code. This is worth emphasizing: the same canonical structure that allows providers to describe their offerings, is the same canonical structure for expressing queries on those offerings, which is in turn the same canonical structure for phrasing service invocation, which is the same canonical structure for representing results. Exposition on this point follows.

### The SSWAP protocol

**Figure 2:**
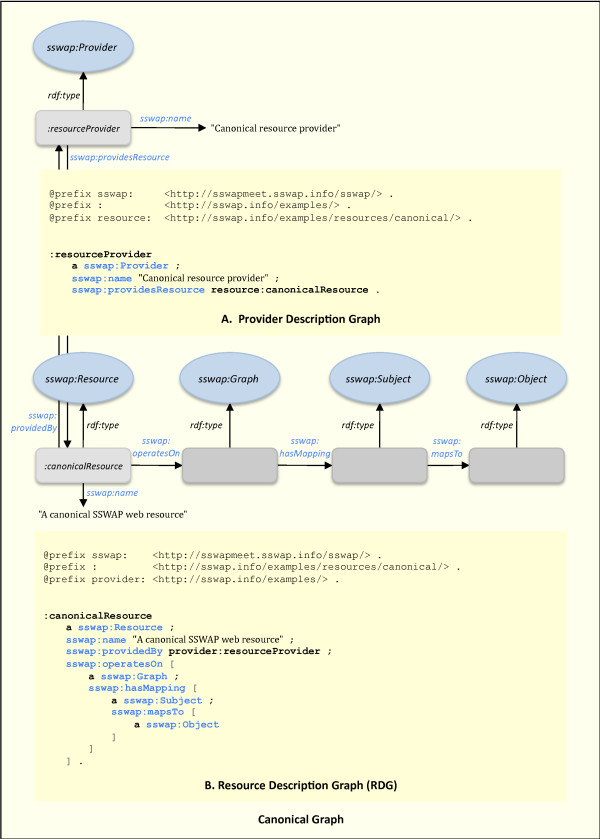
**The Canonical Graph**. OWL DL definitions (not shown) of the fundamental SSWAP classes require all SSWAP compliant resources to express themselves within the framework of the five classes and six predicates as shown here. General practice is to create one file for the provider's description (2A), and one file for each semantic web service (2B). Ovals represent classes; blue ovals are SSWAP reserved classes, orange ovals are third-party classes; rounded-cornered rectangles represent instances (individuals); arrows represent predicates (properties). SSWAP uses the W3C sanctioned encoding of RDF/XML, but code snippets shown here and in the following figures is in the more compact N3 solely for readability. A note on N3: the word '*a*' is shorthand for *rdf:type *and denotes instantiation. Multiple classes are specified serially in a statement with a comma (,) delimiter. A semicolon (;) means that the RDF subject of the assertion is the same as the subject of the preceding line, so the line lists only the predicate and object; blank nodes are represented by square brackets ([]) which allow a nesting of relationships. In some cases blank nodes are labeled explicitly using the prefix underscore-colon (_:) followed by the label.

SSWAP deploys semantic web technologies in a more restricted manner than the broad scope of the semantic web proper. By doing so, it is compatible with the web and web server practices, but does not lay claim to the broader visions of the semantic web. Specifically, providers describe their resources to the world by putting an OWL DL RDF/XML document on the web available to anyone via a simple HTTP GET. This means that anyone can read a resource's description by simply dereferencing its URI. The document, called a Resource Description Graph (RDG), makes specific statements about the resource by using a subset of the SSWAP protocol (see the Annotated SSWAP Protocol page at ). The protocol defines a small set of reserved OWL classes and predicates that allow any provider to describe itself (Figure 2a) and its resources (Figure 2b) within a canonical, recognizable structure. In practice, service providers host one RDF/XML file defining themselves as a *sswap:Provider*, and any number of other RDF/XML files, each with its own *sswap:Resource*. Of course there is no implementation requirement that these be physical files: *e.g*., web masters may deploy servlets to parse URIs. The explicit use of reserved classes and properties allows us to establish an explicit semantics under the larger umbrella of OWL DL. This means machines have clear, unambiguous rules within which to parse any SSWAP graph, and can ignore assertions that are not relevant to SSWAP. Out of a universe of ways one could use OWL DL to describe a resource, the canonical graph structures all resource definitions in terms of a transformation of some (possibly null) input to some (possibly null) output. The protocol delivers a minimal set of constructs to allow providers to describe their resources, and for resources to assert their data/service transformations. Implementers may use OWL DL to add and extend properties and classes and further idiosyncratically describe their offerings. We are currently examining the feasibility of front-ending RDGs with RDFa [28], such that providers could simply host marked-up web pages that could be transformed into RDGs upon the action of an agent. This would tighten SSWAP's position with linked data [29].

In Figure 2A, the canonical graph states that there is some provider (*i.e*., *:resourceProvider*), and out of the universe of relationships it has to things in the world (*i.e*., additional RDF subject-predicate-object assertions not shown on the graph and immaterial to SSWAP), it has a particular *sswap:providesResource *relationship to another individual of type *sswap:Resource*. Here, individuals are web resources. SSWAP guarantees that dereferencing any node with a *sswap: *prefix via a HTTP GET will return a SSWAP-compliant, OWL DL definition of the node which defines appropriate restrictions, cardinality constraints on properties, and so forth. Indeed, SSWAP best-operating practices state that any URI referencing an ontological term in a SSWAP-compliant graph should always be dereferenceable, returning an OWL DL definition of the resource. If a URI is not dereferenceable, the system does not fail, but actors will simply not be privy to on-demand resolution of a term's definition. This works well with OWL's guarantee of monotonicity. In this manner, the architecture brings the flavor and power of hyperlinks and linked data to web service interface declarations. In Figure 2B, the URI *:canonicalResource *reciprocates the association assertion back to the provider with a *sswap:providedBy *predicate. This reciprocation, along with a default behavior, is used by the platform to enforce bilateral assertions so that, for example, a resource's claim to be provided by a third-party provider can be verified by querying the provider. The resource also has a *sswap:operatesOn *relationship to a *sswap:Graph*. The *sswap:Graph *class allows one to build data structures so providers and clients can unambiguously distinguish between such structures as a list of pairs and a pair of lists. The blank node (an anonymous resource) that is a *sswap:Graph *may also have many relationships to other things in the world, but again, we are interested only in its *sswap:hasMappping *relationship to a *sswap:Subject *which in turn is related by *sswap:mapsTo *to a *sswap:Object*.

The *sswap:Subject *and *sswap:Object *classes are used to identify input to output mappings, and as such are equivalent to the input parameters and output return values of traditional interface declarations. For example, Soybase, Gramene, and LIS (Legume Information System) SSWAP services all use the value associated with their various *sswap:Subject*s as lookup keys into databases from which they return a value associated with the *sswap:Object*. But the semantics of the *sswap:mapsTo *predicate does not force a delineation of input and output data: RDF's support for multiple relationships means that it offers a natural way for providers to specify one-to-many and inverse mappings (*e.g*., given a *sswap:Object*, return a *sswap:Subject *by reversing the subject and object of the *sswap:mapsTo *relation). Because SSWAP does not use traditional, ordered input and output parameters, invocation requirements can be changed, deleted, or appended within the constraints of a first-order logic amenable to machine-reasoning, thereby alleviating one of the major disadvantages of traditional, static interfaces. SSWAP builds a semantic web service interface based upon logical assertions, thus delivering an enabling environment whereby machines can better reason at transaction time to assess suitability-for-purpose.

### The Web of Ontologies

**Figure 3:**
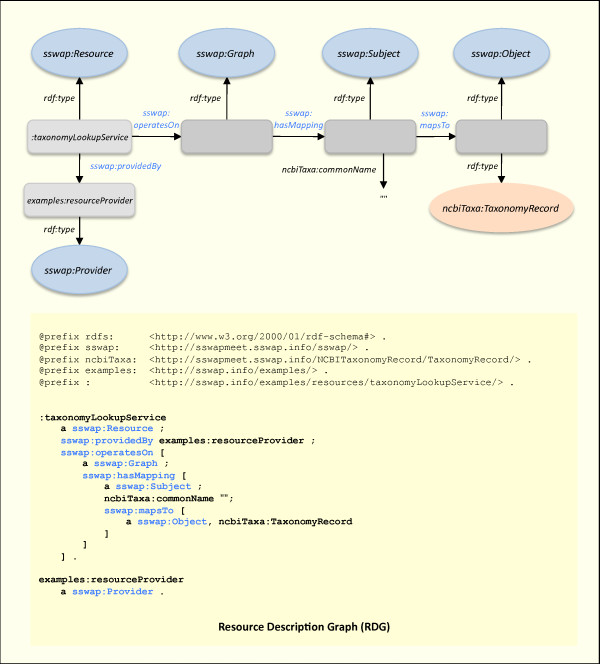
**Resource Description Graph (RDG)**. An example of how the semantic web service *taxonomyLookupService *describes itself using SSWAP classes and predicates. In this case, the resource maps any *sswap:Subject *with a *ncbiTaxa:commonName *to a *sswap:Object *of type *ncbiTaxa:TaxonomyRecord*. This is an example of using a database key (a string) to retrieve data. The full Resource Definition Graph (RDG) is at  and is available to anyone via a HTTP GET.

Figure 3 shows how a resource customizes the canonical graph to describe the specific web resource (service) it is offering to the world. The resource does this by qualifying the nodes that are instances of *sswap:Resource*, *sswap:Graph*, *sswap:Subject*, and *sswap:Object*. In Figure 3, the *taxonomyLookupService *states that it maps the string value of the predicate *ncbiTaxa:commonName *to an object of type *ncbiTaxa:TaxonomyRecord*. This service is a working example  addressing a minor but recurring problem in bioinformatics: the problem of unambiguously and universally associating data with the species or taxon from which they were derived. For human readable web pages and idiosyncratic data files, this is traditionally done by inserting *ad hoc *strings naming the taxon or hyperlinks to database entries. Yet the non-standardized nature of these common sense conventions makes it almost impossible to write broadly re-usable code to reliably extract this metadata across generic web sites. "What data is currently available on legumes?" is a laborious and difficult question to answer. Even seemingly trivial complications such as spellings and alternative names for taxa offer substantial challenges to writing generic, high throughput parsers. These types of tower-of-Babel problems occur regularly in bioinformatics and they conspire to thwart integration. RDF offers a natural mechanism to address a proximal solution: simply reference an unique URI for each taxon and then associate the URI whenever we wish to reference the taxon. For example, the persistent and unique URI for the legume *Medicago truncatula *at NCBI's Taxonomy database is . When we associate data with this URI under a well defined semantic, we are making a machine-parseable, unambiguous assertion that can be used to universally tag the data with the taxon. The *taxonomyLookupService *accepts a string of common words as a key (*e.g*., the common name "barrel medic") and queries NCBI for the associated taxonomy record (*Medicago truncatula*). If it finds a record, it returns the taxon's URI as well as supporting properties as defined at *ncbiTaxa:TaxonomyRecord*.

Ontologies are systems of terms and their relationships. In this example the *taxonomyLookupService *service is using terms from the third-party ontology *ncbiTaxa *for both the *sswap:Subject *and *sswap:Object*. Yet generically SSWAP allows a mix-and-match of terms from across ontologies on the web. This support for mixing independent third-party ontologies while under a formal semantic is central to SSWAP's sociological model of achieving a shared semantic (see ). This offers an important distinction from traditional XML/DTD-based models. In those cases, the lack of a formal semantic and logic underlying the data model and tagging scheme means that extensions tend to break the standard. In SSWAP, OWL's formal first-order description logic (DL) means that extensions are amenable to machine reasoning. In practice, implementations of first-order DL reasoning lags behind our ability to make assertions, so SSWAP relies heavily on basic subsumption and realization (including inferences from rdfs:domain and rdfs:range). Interestingly, it is not necessary that there exist a formal ontological mapping between any two ontological concepts--a problem that in its generic form of ontology alignment is difficult and unsolved. The sole existence of a resource description graph (RDG) is a *de facto *claim of at least a partial ontology alignment: it is a statement to the world that this resource, offered by this provider, offers a service that maps instances of one ontological concept to another. The appropriateness of the mapping may be further described within OWL DL, or may lie implicit in the resource's implementation.

Because all providers and clients have public access via URIs to the same set of ontological terms on the web, the semantics of data and services is open to a shared evolution and marketplace competition. Well-defined, useful terms maintained by trusted public resources encourage re-use. This forms the basis of a shared semantic and consequent integration. Integration is further enhanced by SSWAP's reliance on OWL semantics, so, for example, one may exploit subsumption relations to deduce suitability-for-purpose rather than relying on lexical matching to enforce naïve equivalency via re-use of the same term. The architecture creates what we call a "Web of Ontologies" that is specifically aimed at refocusing the labor of ontology construction from monolithic enterprises to distributed, dynamic "atoms of knowledge."

In summary, SSWAP encourages third-parties to build ontologies as they see the world. It then supplies a framework whereby anyone can pick and choose which concepts best fit what they are looking for or what they are offering while allowing providers to associate these concepts with data types. It additionally supplies a mechanism to "learn more"--that is, enable semantic negotiation--about unrecognized terms by performing an HTTP GET on any term to get a graph of OWL DL statements about the term, be the resource either a service or an ontology term.

### Resource publication with a Discovery Server

A provider defines its resource by putting the SSWAP-compliant, OWL DL resource description graph (an RDG) on the web, accessible to anybody by a simple HTTP GET. In this manner, providers describe themselves in the web, as part of the web, and use the web not just as a data delivery pipeline, but with all its associated infrastructure. Architecturally, this means their "publishing of service" consists of no more than placing an SSWAP OWL DL RDF/XML document on a web site and supporting a HTTP POST (or HTTP GET w/a query substring) for service invocation. The page can be deployed using their organization's standard web practice work flow; it can be changed without coordination with a central site, and so forth.

In practice, we do not run spiders crawling the web to find SSWAP resources, so to garner publishable resources, we run a semantic discovery server at  that hosts a URL http://sswap.info/publish-resource that accepts HTTP POSTs from resources to inform the discovery server of their presence. (Alternatively, humans may interactively publish resources at .) Because resource description graphs sit on the web like any other document, there is no active registration process with the discovery server, just like there is no active registration process with Google. This alleviates many of the security issues associated with de-registration or changing service definitions associated with active registration models, though it also means that the discovery server's knowledge of resources may be out of date with what is currently live on the web. When the discovery server is informed of a resource, it will at its discretion perform a HTTP GET on the resource to retrieve its description graph. Upon retrieving the RDG, the discovery server dereferences terms up to three levels of indirection in an attempt to broaden its knowledge of concepts (ontology terms) used by the resource description graph (RDG). We refer to the resulting set of RDF statements as a three degree closure. We then validate the graph for SSWAP consistency using the OWL reasoner Pellet [30]. We use Pellet to make explicit any and all implicit statements. The reasoner performs the following four operations: *i*) classification: computing all subclass relations between named classes, *ii*) realization: assigning individuals to their most specific subclass, *iii*) consistency checking: complete check for logical contradictions, and *iv*) satisfiability checking: complete check for classes empty by necessity.

As an example, the *taxonomyLookupService *accepts as input an object with the predicate *ncbiTaxa:commonName *(data type *xsd:string*). This allows one to query the service with a common taxon name as a lookup key to the official NCBI taxon web page and scientific name. When building a knowledge base (KB) of resources, our reasoner executes a HTTP GET on *ncbiTaxa:commonName *and examines its definition. It discovers that the domain of the predicate *ncbiTaxa:commonName *is *ncbiTaxa:TaxonomyRecord*--an ontological class tagging the object. The reasoner then correctly classifies the *taxonomyLookupService *service as accepting objects of type *ncbiTaxa:TaxonomyRecord*, even though the service definition never made the statement explicitly. This inference step generates explicit sub-class and sub-property relationships from implicit relations that broaden the KB's statements for later semantic searching. In Kantian terms this is the generation of *synthetic a priori judgments *and constitutes new knowledge [31]. In practice, a RDG of a dozen or so statements is often rendered into thousands of statements after execution of the third degree closure and reasoning (try it at ). This reasoning step greatly enhances capabilities for semantic searching.

If a resource is already in the KB and the URI dereferences to an invalid description graph, then the resource is flagged for removal from the KB; similarly, if the graph is new or changed, then the discovery server updates its internal model appropriately. Currently this is done by rebuilding the entire KB because algorithms for making incremental changes to first-order description logic knowledge bases are still in their infancy. Once we have built a KB, we use the open source applications of Jena [32] for RDF manipulation via SPARQL on top of a PostgreSQL RDBMS [33] as a triple-store.

### Resource Discovery and Semantic searching

**Figure 4:**
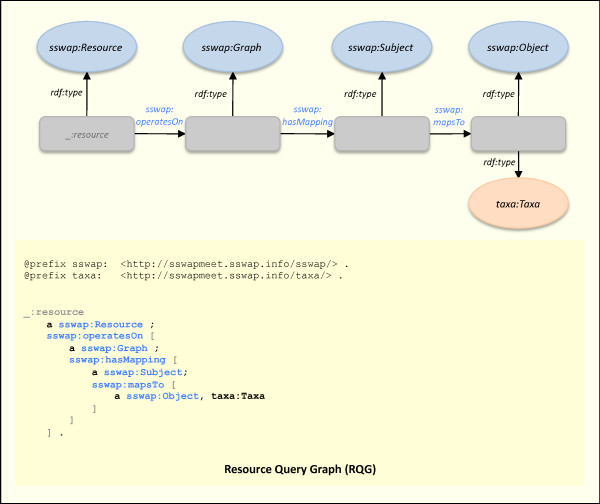
**Resource Query Graph (RQG)**. Clients query a discovery server for data and service providers using the same canonical graph structure and sharing the same publicly available ontologies used by providers when the providers described their resources. The query in the figure is asking "Get me all resources that map anything to a *taxa:Taxa*". Anyone can do a HTTP GET on *taxa:Taxa *to see its definition. Architecturally, more complicated queries can be built by using additional OWL DL assertions, replacing blank nodes with individuals, or using concepts from other publicly available ontologies. Currently, the discovery server running at  does not support all OWL DL embellishments but implements semantic searching by returning all services that are subclasses of the *sswap:Resource *with super classes of the *sswap:Subject *and subclasses of the *sswap:Object*. For programmatic access see .

Clients engage the discovery service by using either the web front-end at  or programmatically engaging the SSWAP query service with a Resource Query Graph (RQG) (Figure 4). Clients construct their query graphs using the same publicly available ontological concepts as used by providers to describe their resource. Just as search engines on the web provide non-exclusive points of entry for web surfing, the discovery server provides a non-exclusive point of entry for resource discovery. Architecturally, clients are not required to use the discovery server; indeed, if they know of a resource's URI then they may engage it directly (see below). In Figure 4, the client is asking the discovery server to return all resources (the named *_:resource *blank node) that map "anything" (the blank *sswap:Subject *node) to something of the class *taxa:Taxa*. A dereference on the *taxa:Taxa *URL gives necessary and sufficient conditions (properties and classes) of individuals of that class. The use of a query graph that has the same canonical structure as description graphs, invocation graphs, and response graphs is a novel approach to operational integration (Figure 1). For example, the client could send a graph without the *taxa:Taxa *qualifier, thereby asking "Get me anyone who can map anything to anything." Clearly, numerous combinations are possible. While the discovery server fulfills the role described variously as "matchmaking" or "brokering" [34], the underlying OWL ontology extends the usual lexical basis for such matches by allowing for semantic searching based on subsumption relations.

For semantic searching, we seek a mechanism to find all resources (services) that are: 1) of a particular (or more specific) type according to some ontology; 2) operate on a particular (or more general) type of data; and/or 3) return a particular (or more specific) type of data. For example, we may want to search for all services that perform sequence comparison, or operate on DNA sequences, or return gene annotations. Consider class *E *defined such that all individuals of class *E *have properties *p *and *q*. We now consider class *V subClassOf E*; necessarily all individuals of class *V *have properties *p *and *q*, and possibly additional properties. This is guaranteed by the formal semantics of rdfs:subClassOf. Thus if we have data belonging to class *V *and we ask the question: "What services can operate on my data?", we should get all services that accept a *sswap:Subject *of type *V *as well as type *E*. Indeed, a service operating on data of class *E *will necessarily work on our data, even if it was constructed independently and in ignorance of class *V*, and even if our data was classified ignorant of class *E *(*i.e*., classifying our data as of class *V *implies that we satisfy its definition, but it does not require that we have knowledge of the complete subsumption hierarchy).

**Figure 5:**
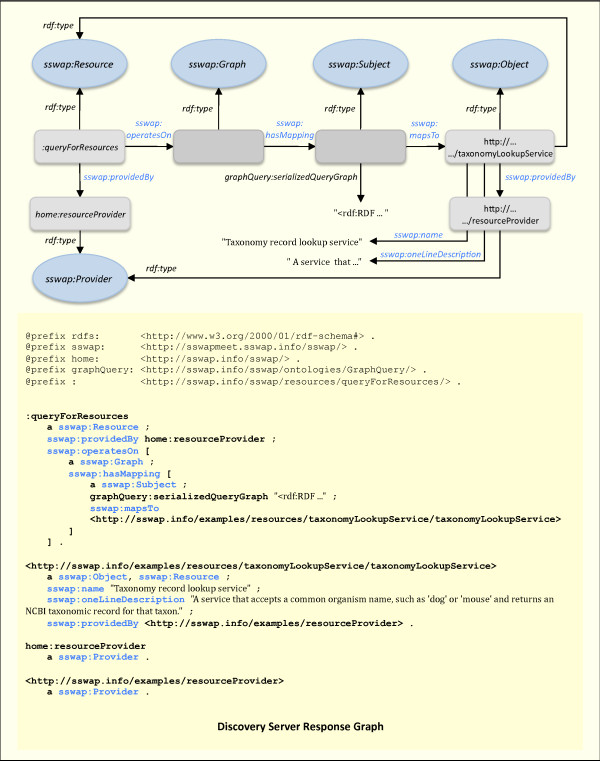
**Discovery Server Response Graph**. The discovery server returns all graphs that satisfy the query. "Satisfaction" means that any resource returned in a response graph could be substituted as an individual of type *sswap:Resource *in the query graph while maintaining the truth of the assertions. Properties of semantic searching (Figure 4) mean that when querying for resources, discovered services are exactly those that are semantically guaranteed to operate on the input data (including those that accept more general classes) while returning the stipulated output data classes (including more specific classes). In the example here the client asked for all services that return data of type *taxa:Taxa *(Figure 4). The discovery server returned a service that returns data of type *ncbiTaxa:TaxonomyRecord*, which is a subclass (a specialization) of *taxa:Taxa*, thereby satisfying the request. Notice how this allows clients to find data and services based on the semantic and ontological relationships of services and data, even if neither the client nor any provider anticipated this usage. Full information is not returned in the response graph, but it available to clients by dereferencing resources to obtain their RDGs.

Figures 4 and 5 give a demonstrative example of how these operational guarantees deliver semantic search results in a decentralized model. In Figure 4., the client asks for all resources that return data of type *taxa:Taxa*. In Figure 5, the discovery server returns a graph with the resource http://...taxonomyLookupService. Dereferencing *:taxonomyLookupService *(retrieving its SSWAP RDG with a HTTP GET) shows that the service returns data of type *ncbiTaxa:TaxonomyRecord *(not shown in figure). Dereferencing that class shows that it is a subclass of *taxa:Taxa*, thereby satisfying the search request based on the semantic relations of the data, not on any lexical equivalencies.

The model of semantic searching with query graphs is to return all resources that are a "sub-concept" of the query graph. Given a query graph with a *sswap:Resource *node of arbitrary named classes *R*_1_, ... *R*_*n*_, with a *sswap:Subject *node of arbitrary named classes *S*_1_, ... *S*_*m *_and a *sswap:Object *node of arbitrary named classes *O*_1_, ... *O*_*o*_, the discovery server returns all known resources that satisfy membership in the class{∩: *R*_1_, ... *R*_*n*_} (and by implication any of its subclasses) with subjects that satisfy membership in any superclass of {∩:*S*_1_, ... *S*_*m*_} and objects that satisfy membership in the class {∩:*O*_1_, ... *O*_*o*_} (and by implication any of its subclasses). As is standard, a class is always a trivial subclass of itself. Thus the returned resources would be (possible) specializations of what one requested; that operate on the typed input data (or generalizations of it), and returned the typed output data (or specializations of it). In this manner, semantic searching seeks to reduce false positives in service discovery by returning those, and only those, services that are guaranteed to operate on the requested data and return transformations that are at least as specialized as requested. In Figure 5, the discovery server response graph sent back to the querying client returns the *taxonomyLookupService *as satisfying the request. The service returns data of type *ncbiTaxa:TaxonomyRecord *(Figure 3) which is a subclass (a specialization) of the requested *taxa:Taxa *in Figure 4. In practice, transaction-time reasoning on graphs to compute subconcepts is expensive and algorithms are still in their infancy. Thus SSWAP satisfies mostly subsumption support on named, explicit classes already established in the KB.

### Service invocation

**Figure 6:**
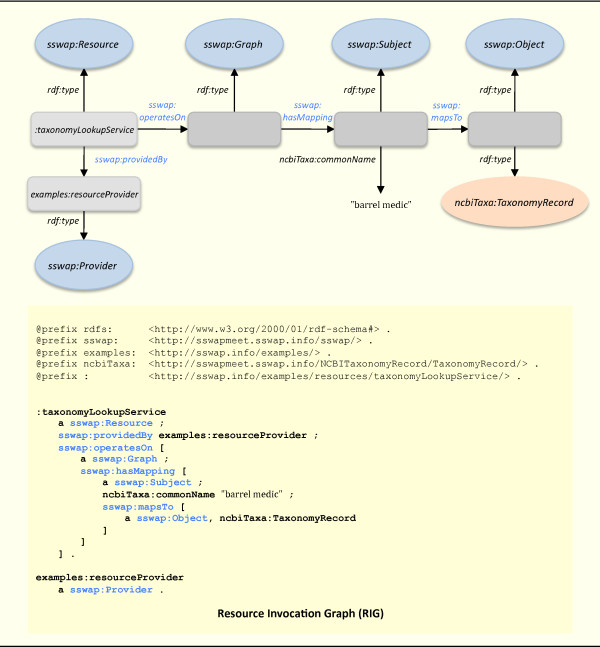
**Resource Invocation Graph (RIG)**. The client creates an invocation graph by either using the response graph returned from the discovery server or executing a HTTP GET on a resource returned by the discovery server to get the resource's latest definition. In either case, the client then annotates the graph with its specific input data. Here, the client enters the key value *ncbiTaxa:commonName *= "barrel medic" and HTTP POSTs the graph to the resource. SSWAP best practices encourage providers to attempt to satisfy invocation graphs sent to them by altering those graphs (for example, by completing *sswap:Subject *to *sswap:Object *mappings) instead of returning their own *de novo *representations of the mappings. This allows clients to assimilate contributions from various resources on a single graph of their choosing by passing it from resource to resource.

By SSWAP convention, POSTing a graph to a resource is interpreted as a request to invoke the service. Once a client has a service's URI (for example, from a discovery server query response graph or its own listings), the client can POST the service's RDG back to the service with input data typed as the *sswap:Subject *(Figure 6). The client always knows the service's interface because the RDG is a logical description of the service's transformation available to anyone with a simple HTTP GET on the same URI used for invocation. In Figure 6, the client replaces the null string value of the predicate *taxa:commonName *with the look up key "barrel medic" and POSTs the graph to the *taxonomyLookupService*. In all cases, the client will either instantiate the *sswap:Subject *with a resource (a URI) that is used as the "input data", or will fill in predicate value(s) directly in the graph (Figure 6).

### Service completion

**Figure 7:**
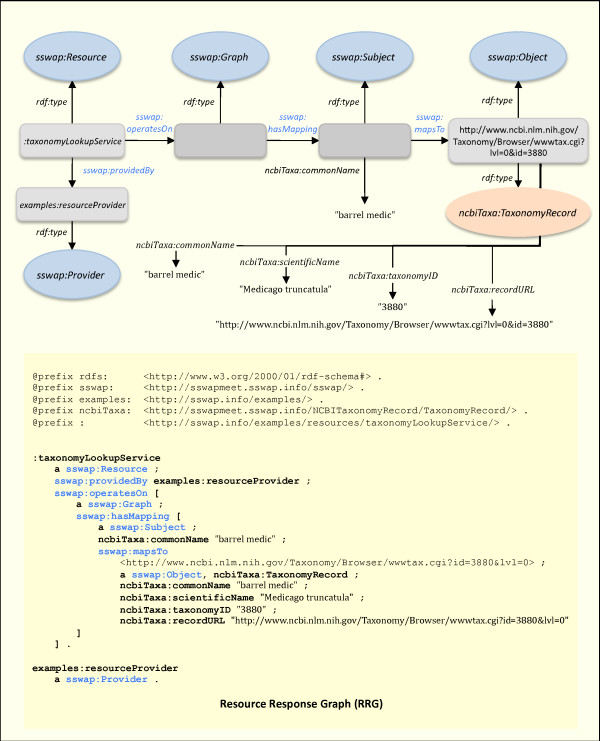
**Resource Response Graph (RRG)**. The graph returned from the resource to the client with the completed *ncbiTaxa:commonName *to *ncbiTaxa:TaxonomyRecord *mapping. In this example the returned *sswap:Object *is an annotated URI: it is both the URI of the *Medicago truncatula *web page at NCBI and has semantically well defined predicates with relevant metadata. Elegant models can be built by returning individuals with URIs pointing to data instead of encoding data directly in the graph. Note how the provider's response (RRG) is a variation on the client's invocation (RIG), which is itself a variation on the provider's description (RDG); all graphs are simple variations on the canonical (Figure 2), including the discovery of new resources (RQG).

The resource provider receives the graph from the client, parses it according to the canonical structure, and looks for ways to enhance it. In Figure 7 the service completes the mapping of the string "barrel medic" to the resource . In this case, the URL is itself a reference to lexical (non-semantic) "output data": *i.e*., a web page. The class *ncbiTaxa:TaxonomyRecord *is defined via various properties that allow for semantically tagging key data such as the scientific name, taxonomy id, etc The service extracts this unstructured information and places it in a structured, semantic context of OWL predicates (*e.g*., *nbciTaxa:commonName*, *ncbiTaxa:scientificName*, etc.).

Upon receiving the response graph from the provider, the client can parse the graph according to its unambiguous structure. Because RDF triples can be parsed order-independent, the client is not required to traverse the graph in a particular hierarchical order. Both content and metadata are "first class" elements, which can be sorted or otherwise searched and organized by clients without losing the provider's statements about the data's relational structure. This helps address the limitations of confounding data content with data structure. To disambiguate content and presentation, SSWAP provides two predicates (*sswap:inputURI *and *sswap:outputURI*) for *sswap:Resource*s which allow resources to identify URIs that should handle the resources' human readable input and output interfaces (see example at ). The combination of RDF order-independence and resource binding to presentation managers, means that SSWAP exists as a true semantic middle layer, appropriate and capable for machine-machine semantic integration.

The canonical graph structures a process for semantic negotiation between client and provider, which is essentially brokered by using shared, third-party ontological terms, the meaning of which are available at transaction time. The document-centric, serialization of a logic (OWL DL) means that the graph can sit in persistent storage without loss of its description/query/answer information. This preserves the integrity of the "data as a statement" and decouples it from the particulars of the current manipulating technology. If in the future a new serialization or a more powerful logic is implemented, then forward compatibility is achieved by translating the OWL DL serialization into the new representation. A relevant example is the relatively new introduction of OWL 2.0.

## Results and Discussion

SSWAP is a semantic web services architecture and protocol. We designed it because SOAP-based web services do not provide a sufficiently rich semantic framework, while the W3C OWL does not provide a sufficiently rich web services framework. SSWAP uses the W3C standard semantic web technologies of RDF (Resource Description Framework; ), RDFS (RDF Schema; ), XML Schema; ) and OWL (Web Ontology Language; ) over HTTP; it does not use SOAP (Simple Object Access Protocol [acronym now deprecated]; ) and its oft accompanying WSDL (Web Services Description Language; ) formalism, nor does it use UDDI (Universal Description, Discovery and Integration; ) for discovery. We rejected SOAP, WSDL, and UDDI because their heavy web service model did not offer strong support for open semantics and description logic reasoning. Adding an explicit and open semantic on top of SOAP, WSDL, and UDDI added a complexity that offered little advantage over using OWL RDF/XML supported by a DL (Description Logic) reasoner over straight HTTP.

SSWAP employs a loose-coupling, late-binding model using OWL DL semantics to achieve dynamic semantic negotiation between suppliers and users of data and services. In SSWAP we introduce a design whereby a single, canonical structure (a template OWL DL graph) embeds the information for how a provider's resource is described and published to the world, how a client's request is made to the discovery service to find a resource, how that request is satisfied by the discovery service, how the client's query is made to the resource, how the resource's answer is returned to the client, and how the client parses that response. This canonical structure means that *the description frames the query frames the answer *(Figure 1). This model is in stark contrast to most existing models where technologies used for resource description, discovery, querying, invocation, and response may share little in common (*e.g*., compare WSDL for description, to SOAP for invocation, etc.). At first it may seem impossible for the provider's resource description to know anything about the client's anticipated query; or the client's discovery request to share anything with the format of the provider's return data. Yet it is in addressing this in a single, mutable graph that allows us to make progress on the problems of the fatal mutability of interfaces, rigidity and fragility of static subsumption hierarchies, and the confounding of content, structure, and presentation. Surprisingly, these graphs are not long lists of idiosyncratic specifications, but are concise representations of the data and its relationships to the resource using publicly available shared ontologies. We conceptually break apart the ontological use of concepts and the data type of objects, and provide an architecture for a dynamic web of ontologies, in a manner strongly analogous to how HTML documents are hyperlinked to each other and one of the major goals of the W3C OWL effort.

The nature of the graph model means that providers may optionally only partially satisfy a graph. This is in distinction from more traditional web service models where services tend to either work completely or not at all. In the SSWAP model, a single resource may not be able to fully satisfy a graph, but a series of resources may each be able to read the graph, append data, and contribute to an integrated, synthetic "answer." SSWAP best practices encourage resources to: *i*) "Do no harm," *ii*) "Ignore what you don't understand," and *iii*) preserve the logical integrity of the graph. The first point means that resources should attempt to accept input (*sswap:Subject*) as broadly as possible (few properties; high level super classes), and return output (*sswap:Object*) as specific as possible (many properties; low level subclasses). This will give clients the greatest flexibility in constructing pipelines of services. In practice it means that between invocation and response, services should modify a graph in any of three ways: *a*) adding new information and instances of *sswap:Object*, thereby fulfilling the mapping, with allowance for inverse mappings; *b*) adding multiple *sswap:operatesOn*, *sswap:hasMapping *and *sswap:mapsTo *predicates to build 1:many, many:1, and many:many mappings as appropriate; *c*) adding explicit statements that are logically implied but not explicitly present. A resource should not remove statements, even if they are logically redundant, since other actors' parsers that do not perform reasoning may rely on their explicit existence. The second point, "Ignore what you don't understand," means that parsers should pass through assertions that cannot be semantically resolved, rather than dropping them or generating an error. This assures that SSWAP statements augment, but do not preclude, other RDF statements in the graph. Both points imply that resources should strive to complete the graph passed to them and return an amended graph back to the client, rather than generating *de novo *"answer graphs" as a response. The third point, to preserve the logical integrity of the graph, means that no resource is required to process, nor should it return, an ill-formed or inconsistent graph. The extent that a partial match is satisfied is up to the provider: open-ended requests leave much discrepancy to providers, while overly-restrictive requests will yield many providers unable to add any information. Either way, both clients and providers treat a graph as a contract, where passage through a provider's service retains the logical consistency and implication of the invoking graph.

Thus SSWAP allows one to combine two powerful features of graph-based semantic web services. The first is partial graph completion, which means that the same graph can be sent to multiple services, and each can annotate or augment it according it own view of the world. This is conceptually distinct from how most traditional web services are implemented, where complete success or complete failure is often a fundamental design feature. The second feature is how SSWAP's persistent canonical graph structure means that pipelining semantic web services is syntactically trivial: the *sswap:Object *of one provider's response graph becomes the *sswap:Subject *of downstream provider's invocation graph. In this manner a query can traverse the web, both in terms of embellishing a single *sswap:Subject *to *sswap:Object *mapping among numerous providers implementing different aspects of the same mapping, and also pipelining *a sswap:Object *to *sswap:Subject *transitively across providers.

SSWAP's enablement of easily mix-and-matching terms across ontologies is closely aligned with the vision of the semantic web and the design of RDF. But from the perspective of well-formed ontological reasoning, one may be hesitant to re-use ontological terms outside of a closed, well-defined ontology. Inevitably, mixing terms from different ontologies can sacrifice desirable global guarantees and can lead to logical inconsistencies if done haphazardly. This is partially addressed by SSWAP's encouragement of using property-rich, subsumption-poor (*i.e*., shallow) hierarchies, since in such systems dynamic subsumption determination is less prone to the rigidity and fragility of deep, static subsumption classifications. Yet SSWAP also addresses this under an embracement of requiring only *local consistency*. In practice, fragmentary knowledge of ontologies is often sufficient for determining suitability-for-purpose in a transaction between two actors. SSWAP enforces local consistency on resources during their publication to the discovery server, but it does not require global consistency across all uses of a term. (Resources not read into the knowledge base [KB] can function independently as semantic web services, but they will not be discoverable at . This is analogous to how traditional web search engines exercise discretion in deciding which URI's they index.) While SSWAP's discovery server does not require global consistency, at regular intervals we do reason over the entire KB to see if global consistency is achievable. To date, we have always achieved this. The ramifications of failing to achieve global consistency mean that some logically equivalent searches could return contradictory responses. From the perspective of expert systems or ontological science this is undesirable, but from the perspective of a world-wide semantic web it is likely that global consistency as a criterion *should *be abandoned. For example, as users we never know the true false positive or false negative rate of non-semantic search engines such as Google, and we are unsurprised if independent searches on "gene" and "hereditary unit" return non-equivalent search results even if these are considered as equivalent keys under some system of definition. Thus SSWAP aims for a middle ground, whereby it brings a level of formalism and semantics to web resources allowing them to describe their offerings, yet still delivers operational value to users navigating within a world of possible logical inconsistencies. OWL's property of monotonicity and its rejection of the unique name assumption ensures that no mix-and-match strategy breaks the properties of completeness and decidability for consistent models, though computational tractability with finite resources is not guaranteed.

For use in semantic web services, ideally ontologies would be constructed with an emphasis on properties (predicates) instead of deep subsumption relations (class hierarchies). Thus the emphasis should be on shallow subsumption ontologies rich in properties, versus deep subsumption hierarchies built axiomatically on rdfs:subClassOf assertions. This approach provides an opportunity to shift subsumption determination from creation time to closer to transaction time, and thereby enhances ontologies' flexibility for addressing suitability-for-purpose. This is expected to increase the likelihood of term reuse by third-parties outside of the original context, and makes it more likely that independently produced ontologies will retain global consistency after aggregation. We have investigated the use Formal Concept Analysis (FCA) to build just-in-time ontologies [22-24].

### Virtual Plant Information Network (VPIN)

We are deploying SSWAP as the underlying semantic technology for the Virtual Plant Information Network (VPIN). The VPIN consists of semantic web services offered by Gramene , SoyBase (soybase.org), The Legume Information System (LIS; ), and over 2400 database and web server entry points from Nucleic Acids Research (NAR). Services are discoverable at . The NAR entries wrap web sites under the NAR service ontologies, but do not map the input or output data types to data-specific ontological terms. A description of the VPIN's Gramene, SoyBase, and LIS services will appear elsewhere. Here, we introduce just a brief introduction to these services to substantiate SSWAP's real-world implementation. Discovery and invocation of these services is available at .

Gramene's cereal QTL resources are available to the community as semantic web services using SSWAP. The use of semantic web services allows Gramene QTLs to be integrated with comparative mapping data, genomic data, germplasm data, and other information that has been made available via the VPIN. The Gramene QTL database includes QTLs identified for numerous agronomic traits in the grasses (*e.g*., rice, maize and barley). The emphasis is on presenting QTLs with information on both associated traits and a mapped locus on a genetic map. Gramene acts as a provider within the VPIN semantic web services platform to describe QTL data and services, to enable discovery of those resources, to allow partners to share and integrate data and terms, and to invoke those web processes to operate on those data. By using these semantic technologies we go beyond token matching (*e.g*., searching for the string 'QTL' on the web) and open a combination of lexical and semantic searching instead. In this manner, users may find services that operate on formal QTL objects. Gramene offers distinct SSWAP semantic web services for accessing QTLs by accession ID, phenotypic trait symbol, trait name, trait synonym, trait category, species scientific name, species common name, QTL symbol, linkage group, and Trait Ontology ID. Future plans are to improve the current ontology, integrate resources with other existing ontologies to enable integration with other services and add further capabilities as the QTL database is expanded.

SoyBase , is the USDA-ARS public repository for community contributed and professionally curated genetic and genomic data for the soybean *Glycine max *(L.) Merr As part of the comparative genomics activity in the Legume Information System (LIS) we have developed and deployed a number of SSWAP services that provide tools for automatic retrieval of data from SoyBase. Two types of services have currently been designed for SoyBase *Locus *and *QTL *classes. The first service type returns a full report of data contained in the Soybean Breeders Toolbox (the current database for SoyBase) for an input soybean genetic map QTL or Locus symbol, *SoybaseQtlReportService *and *SoybaseLocusReportService *respectively. The second type of services return a selection of data type properties (database fields) making them more atomic and easily parseable. The services deliver data combinations which would most likely to be needed by other databases or soybean researchers, such as the ability to get a list of all soybean germplasms in which a marker has been analyzed (*SoybaseLocusGermplasmService*) or the ability to get a list of all soybean genetic maps to which a SoyBase *Locus *is associated. Other databases can interrogate the Soybean Breeders Toolbox and retrieve a list of the types of soybean loci available (*SoybaseLocusTypeService*). This list can then be used to systematically retrieve all loci from the database by their "type" using the *SoybaseLocusByTypeService *and so on. Future improvements to the SoyBase SSWAP services will include the ability to retrieve sequence data associated with soybean loci as well as the expansion of services to include other data types contained in the Soybean Breeders Toolbox. As the soybean genomic sequence data becomes publicly available and incorporated into the Toolbox, information on those data will also be made available through SoyBase SSWAP services.

The Legume Information System (LIS) has developed SSWAP semantic web services in collaboration with Gramene and SoyBase. First generation services provide an entry point to the Legume Information Network (LIN), while second generation services return LIS genomic and transcriptome sequences given accession ids and marker symbols, as well as a service entry point to BLAST. The LIS service *getSequenceForIdentifier *demonstrates the ease in which RDF allows one to return pointers to data instead of embedding large amounts of data in XML files. The service maps a GenBank accession number, a TIGR transcript assembly number, or a TIGR consensus sequence number to its associated LIS sequence. Because sequence data may be voluminous, instead of embedding the data in the response graph, the service makes the *sswap:Object *a URL to where the data is located. It annotates the URL with a class (ontological term) designating it of type FASTA, and appends a property with the FASTA header information. Users of the service simply dereference the URL with an HTTP GET to retrieve the FASTA sequence.

If Gramene, SoyBase, and LIS had used traditional web service technologies, their offerings would have proliferated the current silo effect now seen with hundreds of web services each requiring low-throughput, non-semantic discovery and engagement. Their use of SSWAP mitigated this by establishing an early ground in providing semantically enabled services and data amenable to semantic discovery and invocation.

## Conclusion

Implementing SSWAP for the VPIN showed both the strengths and limitations of the semantic web services approach. Re strengths, the ontological flexibility of the model (the "Web of Ontologies") means that new data types and service categories can be introduced in a manner that makes them approachable and available to all actors. In contrast to the flexibility of data and service descriptions, SSWAP protocol standardization means that parsing and low-level code can be easily shared across implementations. Standardization over a formal semantic means that we can apply a reasoner to discover implicit truths, and then build a semantic search capability to use those assertions to return services based on both their service categorization and their data's semantic tagging, as well implied subsumption relations. In future implementations, the deployment of reasoners not only for KB building supporting semantic searching but also at transaction time by individual actors offers a promising approach for addressing the fatal mutability of traditional interfaces. When combined with property-rich classes and shallow subsumption relations, the approach alleviates many of the limitations of static subsumption hierarchies. Finally, OWL's reliance on RDF and our use of SSWAP predicates such as *sswap:inputURI *and *sswap:outputURI *provides a natural mechanism to segregate data and content from presentation layers.

Yet our research also found limitations: 1) OWL DL's property of monotonicity means that some simple properties of web services, such as specifying and enforcing the number and/or codependency of required and optional parameters for a service can be obtuse (*e.g*., specifying BLAST parameters). Work-arounds address this with a best practices convention on the use of necessary and sufficient owl:Restrictions (*e.g*., see ) but the fit is not natural from a web services perspective; 2) a service's logical description can be implemented in any number of different syntactical ways. Thus for validation, it is not sufficient to parse graphs solely based on syntactical structure. Yet just-in-time reasoners deployed at transaction time are not well developed, so currently service providers rely heavily on parsing explicit statements which may lead them to incorrectly accept or reject some invocation graphs; 3) OWL DL may be too powerful. Complex restrictions on classes, predicates, and individuals can be verbose and difficult for humans to master when serialized in RDF/XML. The recommended approach is to work at a higher level employing reasoners and parsers to operate on the underlying graphs. But reasoners are still maturing and have some undesirable properties. For example, it is difficult or even impossible to estimate how long a reasoner will take to complete based on a given input graph; 4) knowledge bases--the result of running a reasoner on a set of statements and inferring new truths--are fragile to incremental additions, deletions, and changes in the underlying input statements. In general, once information changes, the reasoner has to be re-run on the entire input corpus, which can take a substantial amount of time. Incremental reasoning is an area of active research; 5) developer tools are still in their infancy. For widespread deployment, bioinformaticians will need helper tools, much in the same way that many web developers use high level languages and integrated development environments to generate HTML and server-side web code.

We view these limitations as "growing pains" on a path towards web integration that offers to transcend many of the limitations that currently limit integration. Some limitations, such as a relative unfamiliarity with OWL in the bioinformatic community or the lack of suitable developer tools are addressable. Other limitations, such as advances in just-in-time or incremental reasoning will require new advances in computer science.

## Availability and requirements

**Project name**: SSWAP

**Project home page**: ; source code deposited at 

**Operating system**: Platform independent; implemented on unix

**Programming languages**: Java, OWL, HTML, JSP

**Requirements for Provider Development Kit**: Java 1.5 or higher, ant 1.7 or higher, Tomcat 5.0 or higher, Jena 2.5 or higher. Hosting an independent discovery server also requires Pellet 1.5 or higher and a database backend such as PostgreSQL 8.2. Protégé 3.3 or higher is useful for developing ontologies.

**License**: minor variant on MIT license; see lib/license.txt on main SVN trunk at .

**Any restrictions to use by non-academics**: none beyond general licensing terms; see license for details.

## List of Abbreviations

DL: Description Logic; KB: Knowledge Base; LIS: Legume Information System; OWL: Web Ontology Language; QTL: Quantitative Trait Locus; RDF: Resource Description Framework; RDFS: RDF Schema; RDG: Resource Description Graph; RIG: Resource Invocation Graph; RQG: Resource Query Graph; RRG: Resource Response Graph; SOAP: Simple Object Access Protocol (acronym deprecated); SSWAP: Simple Semantic Web Architecture and Protocol; UDDI: Universal Description Discovery and Integration; VPIN: Virtual Plant Information Network; W3C: World Wide Web Consortium; WSDL: Web Service Definition Language; XML: Extensible Markup Language; XMLS: XML Schema; XSD: XML Schema Document; URI: Uniform Resource Identifier; URL: Uniform Resource Locator.

## Authors' contributions

DDGG was the Principal Investigator on the project and wrote the main paper; GSS was the principal software developer; GDM was co-PI for LIS; SA provided software development for Gramene and wrote the section on Gramene services; CDT was co-PI for JCVI; DG was oversight for SoyBase; RTN provided software development for SoyBase and wrote the section on SoyBase services. All authors read and approved the manuscript.
